# Pyrophosphate levels strongly influence ascorbate and starch content in tomato fruit

**DOI:** 10.3389/fpls.2013.00308

**Published:** 2013-08-09

**Authors:** Sonia Osorio, Adriano Nunes-Nesi, Marina Stratmann, Alisdair R. Fernie

**Affiliations:** Max-Planck-Institut für Molekulare PflanzenphysiologieAm Mühlenberg Potsdam-Golm, Germany

**Keywords:** tomato fruit, ripening, ascorbate, sugars, pyrophosphatase

## Abstract

Ascorbate (vitamin C) deficiency leads to low immunity, scurvy, and other human diseases and is therefore a global health problem. Given that plants are major ascorbate sources for humans, biofortification of this vitamin in our foodstuffs is of considerable importance. Ascorbate is synthetized by a number of alternative pathways: (i) from the glycolytic intermediates D-glucose-6P (the key intermediates are GDP-D-mannose and L-galactose), (ii) from the breakdown of the cell wall polymer pectin which uses the methyl ester of D-galacturonic acid as precursor, and (iii) from *myo*-inositol as precursor via *myo*-inositol oxygenase. We report here the engineering of fruit-specific overexpression of a bacterial pyrophosphatase, which hydrolyzes the inorganic pyrophosphate (PPi) to orthophosphate (Pi). This strategy resulted in increased vitamin C levels up to 2.5-fold in ripe fruit as well as increasing in the major sugars, sucrose, and glucose, yet decreasing the level of starch. When considered together, these finding indicate an intimate linkage between ascorbate and sugar biosynthesis in plants. Moreover, the combined data reveal the importance of PPi metabolism in tomato fruit metabolism and development.

## Introduction

Nutrition is, by definition, aimed at maintaining human cell and organ homeostasis (Goodacre, 2007). In this context, a balance diet should be considered not just to include carbohydrates, proteins, and lipids, but also other physiologically active components such as certain amino acids and vitamins. Plants are the main dietary source in almost all trophic chains. Therefore, human nutritional health is ultimately dependent on the intake of mayor and minor nutrients from plants, especially given that humans are unable to synthesize certain organic compounds such as vitamins (Fitzpatrick et al., 2012).

L-ascorbate (AsA) is commonly called “vitamin C.” In plants, it acts as a scavenger of the free radicals generated by photosynthesis, cellular respiration, and abiotic stresses such as ozone and UV radiation (Conklin et al., 1996; Noctor and Foyer, 1998; Smirnoff and Wheeler, 2000). AsA has additionally been described to play an important role as an enzyme cofactor while participating in defense, cellular elongation, division and fruit ripening (Arrigoni and De Tullio, 2002; Pastori et al., 2003; Green and Fry, 2005). In humans, AsA has an integral role as cofactor of some dioxygenases enzymes which are involved in biosynthesis of carnitine and collagen (Padayatty et al., 2003). Therefore, its deficiency is associated with conditions such as scurvy and low immunity, which is mainly a consequence of the inactivation of these dioxygenases (De Tullio, 2012). AsA has also been associated to molecular events such as oxygen sensing, redox homeostasis, and carcinogenesis (Valko et al., 2006). In addition, different epidemiological studies have established a positive link between AsA content in food and health benefits such as the prevention of cardiovascular disease, cancer, and other inflammatory diseases (Blot et al., 1993; Steinmetz and Potter, 1996). Current approaches to relieve micronutrient deficiencies include the promotion of balanced diets, supplementation and food fortification, such as iodination of salt or fluoride fortification of toothpaste and tap water (Fletcher et al., 2004; Poletti et al., 2004). However, AsA is a difficult micronutrient for food fortification since it is oxidized very easily. AsA biofortification through metabolic engineering therefore represents an attractive alternative strategy to increase the intake of natural AsA in rich and poor countries alike (Muller and Krawinkel, 2005).

In plants, the biosynthetic pathway of AsA occurs by four different pathways, D-mannose/L-galactose (D-Man/L-Gal) or Smirnoff–Wheeler pathway, the major AsA biosynthetic route in plants, which involves GDP-D-mannose in the initial step (Wheeler et al., 1998). An alternative pathway with L-galacturonic acid as intermediate has been reported in strawberry, which proceeds via D-galacturonic acid to L-galactono-1,4-lactone (Agius et al., 2003) that serves as the linkage with the D-Man/L-Gal pathway. There are also alternative pathway of synthesizing AsA through the intermediates of L-gulose (Wolucka and Van Montagu, 2003) and *myo*-inositol (Lorence et al., 2004). However, the *myo*-inositol pathway remains controversial due to the lack of strong evidence. The AsA concentration remains the same in wild type and *MiOX* overexpression lines (Endres and Tenhaken, 2009).

**Figure A1:**
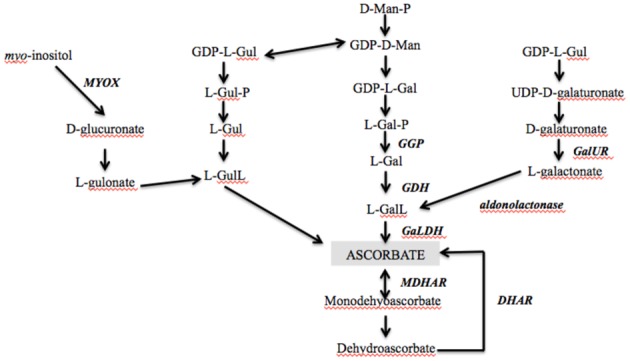
**The proposed biosynthetic pathways for ascorbate in plants**. L-Gal, L-galactose; L-GalL, l-galactono-1,4-lactone; -LGul, L-gulose; L-GuL, L-gulono-1,4-lactone; D-Man, d-mannose; UDP, uridine diphosphate; *GGP*, GDP-L-galactose phosphorylase; *GDH*, L-galactose dehydrogenase; *GaLDH*, L-galactono-1, 4-lactone dehydrogenase; *GalUR*, D-galacturonate reductase *MYOX*, *myo*-inositol oxidase; *MDHAR*, monodehydroascorbate reductase ; *DHAR*, dehydroascorbate reductase.

Formation of GDP-D-mannose is the initial step in the D-Man/L-Gal pathway, which is synthesized from D-mannose-1P via GDP-mannose pyrophosphatase (Conklin et al., 1999) (Figure A1). This reaction generates inorganic pyrophosphate (PPi) as by-product. In plants, PPi plays a central role not only as by-product of activation and polymerization steps (Sonnewald, 1992; Geigenberger et al., 1998; Rojas-Beltran et al., 1999; Farre et al., 2001), if not as an energy donor *per se* (Stitt, 1998; Lopez-Marques et al., 2004). PPi is generally removed by inorganic pyrophosphatases, which hydrolyze PPi to orthophosphate (Pi). Heterologous expression of the *Escherichia coli* pyrophosphatase in an untargeted manner, conferring cytosolic localization of the encoded protein, showed an important role in the partitioning between sucrose (Suc) and starch (Sonnewald, 1992; Farre et al., 2001; Lee et al., 2005). In contrast, expression of the *E. coli* pyrophosphatase targeted to the plastid displayed only minor changes in metabolites levels (Farre et al., 2006). A transient down-regulation of plastid-targeted soluble pyrophosphatase in *Nicotiana benthamiana*, revealed an important role in photosynthesis as well as in the regulation of water exchanges under mild drought stress (George et al., 2010).

In this study, we generated *Solanum lycopersicum* cv MoneyMarker lines, which overexpress the gene encoding the inorganic pyrophosphatase from *E. coli* in an untargeted manner under the control of a fruit specific promoter. This strategy resulted in increased vitamin C levels up to 2.5-fold in ripe fruit as well as increasing in the major sugars, sucrose, and glucose, yet decreasing the level of starch. When considered together, these finding indicate an intimate linkage between ascorbate and sugar biosynthesis in plants.

## Results

### Generation of B33-PPi overexpression tomato lines

**Figure 1:**
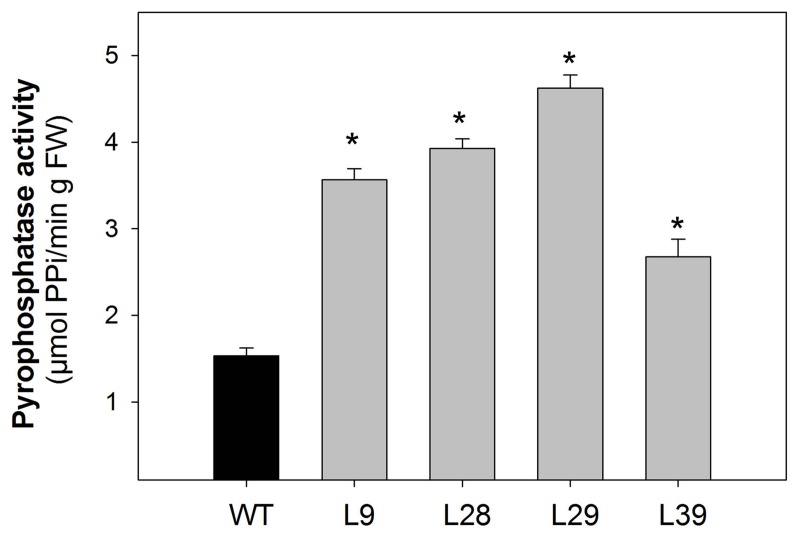
**Pyrophosphatase activity of B33-PPi tomato lines**. Pyrophosphatase activity in red tomato fruit (60 DAP). An asterisk indicates the values that were determined by the *t*-test to be significantly different (*P* < 0.05) from wild type.

To assess the effect of over expression of a pyrophosphatase from *E. coli* (Sonnewald, 1992) in tomato fruit we introduced this gene in the sense orientation under the control of the patatin B33 promoter (Jelitto et al., 1992). This promoter has been shown to be ripening-specific promoter in tomato fruit (Frommer et al., 1994; Centeno et al., 2011). An initial screening was carried out on the basis of pyrophosphatase activity of ripe tomato fruits (data not shown). This screen allowed the identification of four lines displaying considerably elevated activity (L9, L28, L29, and L39), which were taken to the next generation. Eight T2 plants per line were grown in the greenhouse and young leaves (3 weeks old plants) as well as fruits at green (35 days after pollination; DAP) and red (60 DAP) stages were harvested. Assay of alkaline pyrophosphatase activity revealed that the selected lines displayed considerable increase in activity in red fruit (Figure 1). To ensure that this increase of target enzyme activity was restricted to fruits, the activity of the enzyme was additionally tested in young leaves where they were unaltered (10.8 ± 0.2; 11.1 ± 0.5; 11.4 ± 0.4; 10.4 ± 0.3; 10.5 ± 0.5 μmol min^−1^ g^−1^ FW in wild type, L9, L28, L29, and L39, respectively; values are mean ± SE).

### Fruit size and yield

**Figure 2:**
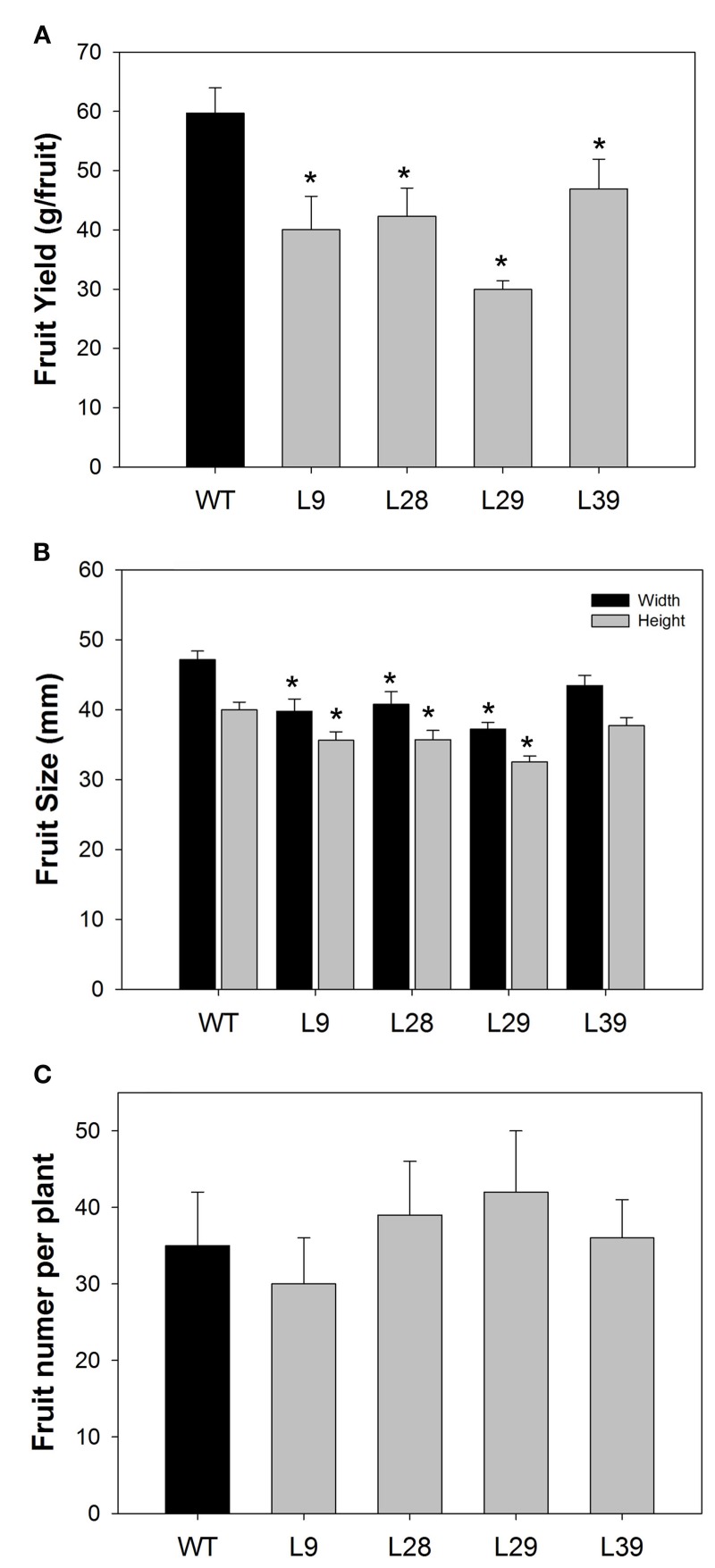
**Characterization of B33-PPi tomato lines**. **(A)** Fruit yield (g per fruit), **(B)** fruit size, and **(C)** total fruit number of B33-PPi lines. All measurements were done in red stage (60 DAP). For all parameters, values are presented as the mean ± SE of eight biological replicates (one biological replicate is represented by one individual plant). An asterisk indicates the values that were determined by the *t*-test to be significantly different (*P* < 0.05) from wild type.

Fruit size and weight per fruit were determined in red fruit (60 DAP). All four lines exhibited a significantly lower weight per fruit (Figure 2A) as well as smaller fruit size in three of the four lines (L9, L28, and L29; Figure 2B). The total fruit number was, however, essentially unaltered (Figure 2C).

### Pyrophosphate and inorganic phosphate levels

Having determined that the transformants displayed the desired alteration in enzyme activity, we next evaluated pyrophosphate levels themselves. For this purpose, pericarp tissues of red fruit at 60 DAP and young leaves (3 weeks old plants) were harvested and pyrophosphate levels were determined taking care to observe all control procedures required to minimize the influence of contaminants (Farre et al., 2001). These analyses revealed significant decreases in pyrophosphate levels in all lines ranking from 25 for L39 to 55% for L29 in red tomato fruit (Table 1). However, as anticipated both from the specificity of expression of the transgene and the lack of change in the activity no changes in pyrophosphate levels were observed in leaves (Table 1). Relatively consistent changes were also seen in the level of inorganic phosphate in red fruit. Inorganic phosphate level increased in three transgenic lines (L9, L28, and L39; Table 1).

**Table 1:** **Pyrophosphate and inorganic phosphate concentration in the B33-PPi red tomato fruits (60 DAP) and leaves (3 weeks old plants)**.

	**WT**	**L9**	**L28**	**L29**	**L39**
	**PPi content (nmol g^−1^ FW^−1^)**				
Fruit	4.3 ± 0.8	****2.5** ± **0.7****	****2.3** ± **0.4****	****1.8** ± **0.6****	****3.2** ± **0.5****
Leaves	12.3 ± 2.5	10.8 ± 2.4	11.5 ± 2.2	13.4 ± 3.5	12.9 ± 2.4
	**Pi content (μ mol g^−1^ FW^−1^)**				
Fruit	3.5 ± 0.5	****7.1** ± **0.7****	****6.9** ± **0.5****	****8.5** ± **0.7****	4.5 ± 0.5

*Values are presented as the mean ± SE of 6 biological determinations. The values that are significantly different by t-test from the wild type are set in bold type (P < 0.05)*.

### Metabolite profiling of green and red fruits of the B33-PPi lines

**Figure 3:**
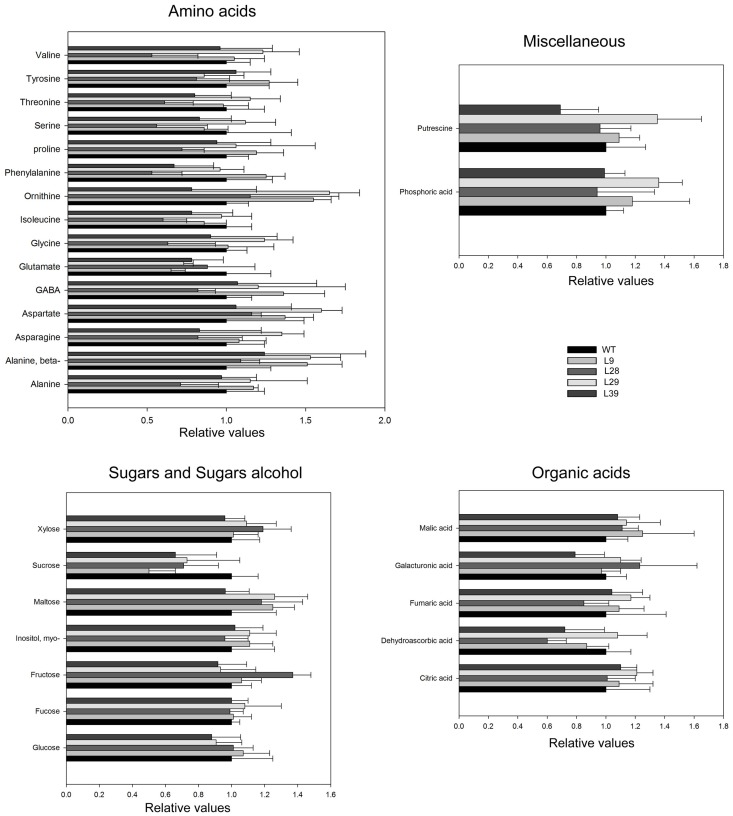
**Primary metabolite levels in the receptacle of WT and B33-PPi lines at green stage**. Data are normalized to the mean value of WT at the G stage. Values are means SE of six replicates. Asterisks indicate significant differences by *t*-test (*P* < 0.01) of the transgenic lines compared with WT at green stage.

**Figure 4:**
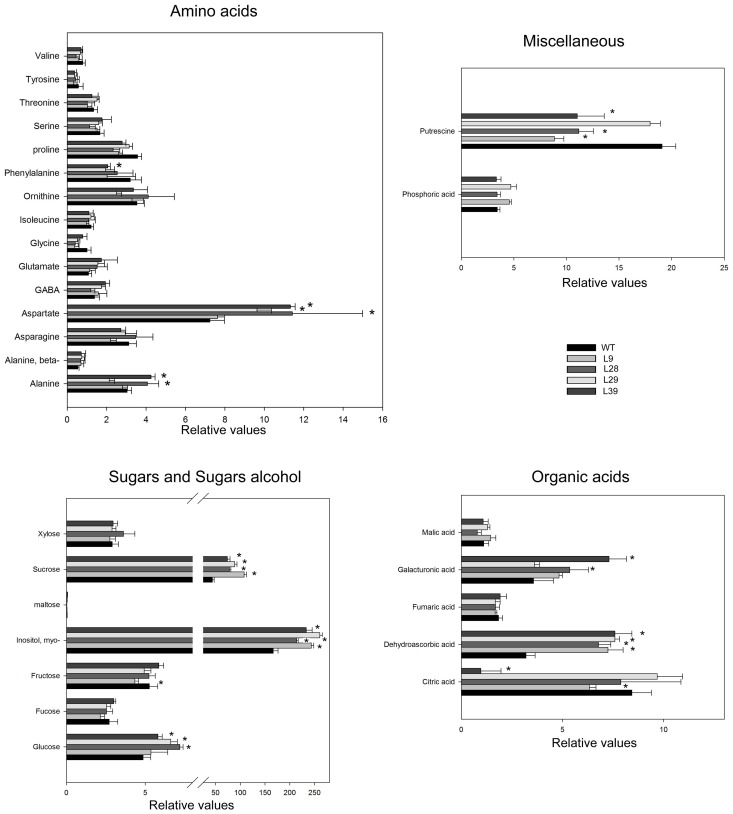
**Primary metabolite levels in the receptacle of WT and B33-PPi lines at red stage**. Data are normalized to the mean value of WT at the G stage. Values are means SE of six replicates. Asterisks indicate significant differences by *t*-test (*P* < 0.01) of the transgenic lines compared with WT at red stage.

In order to further characterize the effects of the reduction of pyrophosphate content, we next applied and established gas chromatography (GC)-MS-based metabolite profiling method (Osorio et al., 2012) to pericarp tissue derived from green and red fruits. Surprisingly, at the green stage the metabolite profiles of the transgenic lines were remarkable similar to those of the WT (Figure 3). However, similar analysis in red stage revealed important changes in the levels of several few metabolites (Figure 4). In all four lines Suc was significantly increased in red tomato fruit by up to 2.5-fold in comparison to WT (Figure 4). This increase in Suc was accompanied by increases in Glc in three lines but no significant changes in Fru (only L9 showed slight decrease). The changes in these sugars were coupled to a decrease in starch content (Table 2) as well as an increase in the total soluble solids content (Brix) in all transgenic red fruit (6.2 ± 0.3; 7.4 ± 0.2; 8.4 ± 0.4; 6.9 ± 0.2 for the wild type, L9, L28, L29, and L39, respectively). To better understand these metabolic alterations, we next measured the AGPase in the red B33-PPi fruits. We observed a decrease in this activity in all transformants with the exception of the L39. However, the activation stage of this enzyme was invariant (Table 3).

**Table 2:** **Starch content in the B33-PPi red tomato fruits (60 DAP)**.

	**WT**	**L9**	**L28**	**L29**	**L39**
	**Starch (nmol g^−1^ FW^−1^)**				
Fruit	438.3 ± 9.5	****354.2** ± **13.1****	****316.4** ± **16.9****	****275.5** ± **20.5****	384.6 ± 27.7

*Values are presented as the mean ± SE of 6 biological determinations. The values that are significantly different by t-test from the wild type are set in bold type (P < 0.05)*.

**Table 3:** **Enzyme activities in the B33-PPi red tomato fruits (60 DAP)**.

**Enzymatic activities**	**WT**	**L9**	**L28**	**L29**	**L39**
	**nmol min^−1^ g^−1^ FW^−1^**				
AGPase	6.65 ± 0.75	****3.98** ± **0.73****	****4.65** ± **0.42****	****3.97** ± **0.89****	6.38 ± 1.02
AGPase activation state (Vsel/Vred)	0.67 ± 0.08	0.58 ± 0.09	0.73 ± 1.04	0.63 ± 0.76	0.76 ± 0.06
MDHAR	6.35 ± 0.31	****12.34** ± **0.21****	****15.22** ± **0.46****	****11.10** ± **0.36****	****9.43** ± **0.42****
GalUR	15.65 ± 0.86	14.89 ± 0.75	16.24 ± 0.65	15.23 ± 0.54	16.33 ± 0.76
Aldonolactonase	11.23 ± 1.34	10.34 ± 0.78	12.36 ± 1.06	13.23 ± 1.03	12.54 ± 0.87
	**μ mol min^−1^ g^−1^ FW^−1^**				
MYOX	12.43 ± 0.93	****8.24** ± **0.85****	****7.77** ± **0.74****	****9.26** ± **0.82****	****10.52** ± **0.69****
GaLDH	0.35 ± 0.09	****0.78** ± **0.07****	****0.97** ± **0.04****	****1.12** ± **0.07****	****0.67** ± **0.05****

*Values are presented as the mean ± SE of 6 biological determinations. The values that are significantly different by t-test from the wild type are set in bold type (P < 0.05)*.

**Figure 5:**
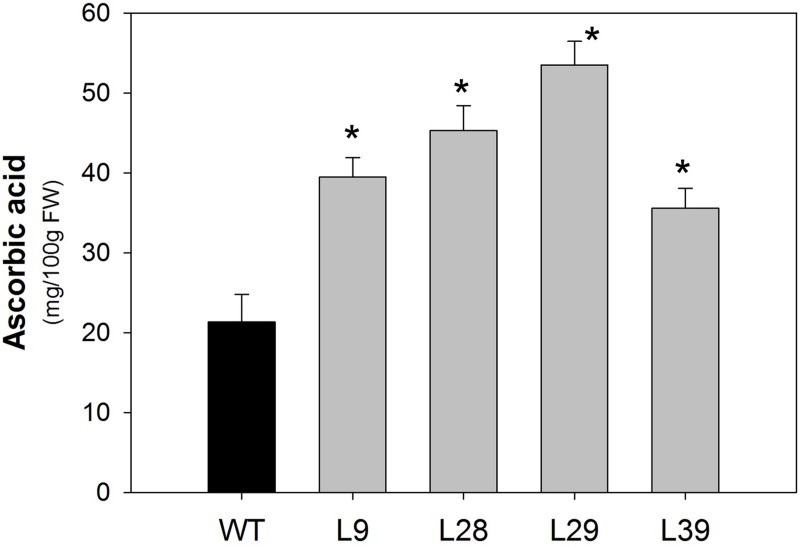
**Ascorbate of B33-PPi tomato lines**. Ascorbic acid was determined in red tomato fruit (60 DAP). The values are presented as the mean ± SE of six biological replicates. An asterisk indicates the values that were determined by the *t*-test to be significantly different (*P* < 0.05) from wild type.

Interestingly, a strong increase in metabolites related to ascorbate biosynthesis, such as dehydroascorbic acid (all four lines), *myo*-inositol (all four lines), galacturonic acid (lines L28 and L39) (Figure 4) as well as a substantial increase of ascorbate (approximately between 2 and 3-fold) were observed (Figure 5).

Additionally, the transformants revealed an increase in two amino acids Ala (L28 and L39), and Asp (L28, L29, and L39) as well as a reduction in putrescine (L9, L28, L39) (Figure 4).

### Expression of *E. Coli* pyrophosphatase in tomato fruits leads to alteration in ascorbic acid biosynthesis

**Figure 6:**
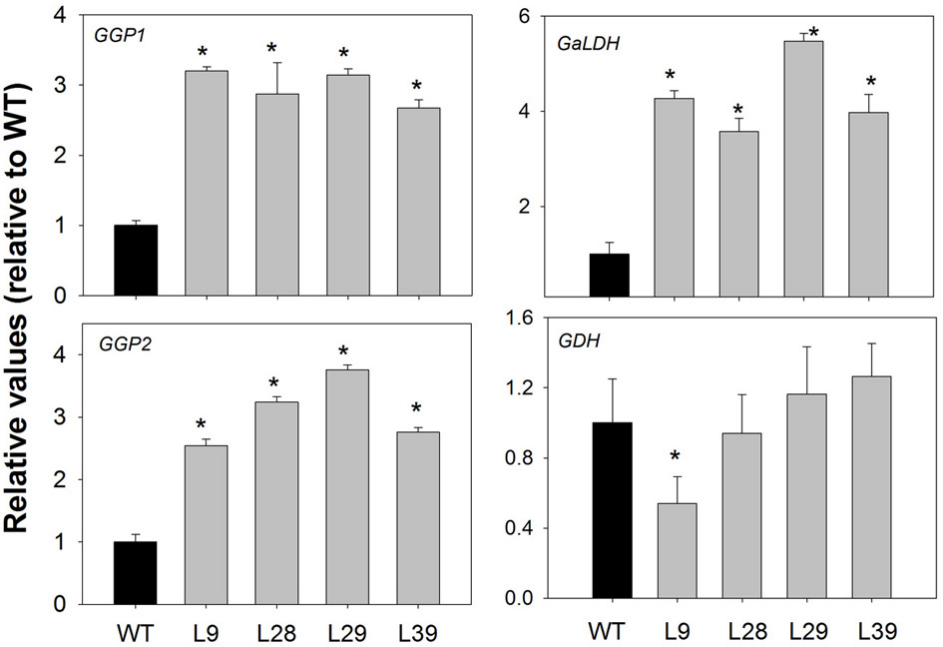
**Expression of *GGP*, *GaLDH*, and *GDH* genes in red B33-PPi fruits**. The abundance of GGP1(acc. number Solyc06g073320), GGP2 (acc. number Solyc02g091510), GaLDH (acc. number Solyc10g079470), and GDH (acc. number Solyc01g106450) mRNAs were measured by quantitative RT-PCR, respectively. An asterisk indicates the values that were determined by the *t*-test to be significantly different (*P* < 0.05) from wild type.

Since some related ascorbate biosynthesis metabolites as well as ascorbate were modified in the red B33-PPi tomato fruits, we next evaluated if ascorbate biosynthesis and/or recycling were altered in these fruits. For this purpose we examined the different AsA biosynthetic pathways. First, we analyzed the transcript levels of some genes in the D-Man/L-Gal pathway. The expression of the two *GDP-L-galactose phosphorylase* (*GGP*) genes, a key point for the control of ascorbate pathway (Dowdle et al., 2007; Laing et al., 2007; Bulley et al., 2012) was up-regulated in all transgenic lines as well as the *L-galactono-1,4-lactone dehydrogenase* (*GaLDH*) gene, while the L-galactose dehydrogenase (*GDH*) gene showed a significant decrease only in one line (L9) (Figure 6). The higher activity of the last enzyme in this pathway, GaLDH, corroborated that D-Man/L-Gal pathway was up-regulated in red ripened B33-PPi fruits (Table 3). Second, related with the D-galacturonic acid pathway, we observed an increase in the level of its precursor, galacturonic acid, in two lines (Loewus and Kelly, 1961; Agius et al., 2003). However, the enzyme activities of the last two enzymes of this pathway, D-galacturonate reductase (GalUR) and aldonolactonase, were unaltered in these fruits (Table 3). Third, we observed that the *myo*-inositol level was altered in all transformants and considering that *myo*-inositol has been proposed as a precursor of ascorbate (Lorence et al., 2004), we determined the total *myo*-inositol oxygenase activity in red fruits (Table 3). Intriguingly, a significant decrease in the total *myo*-inositol oxygenase activity was observed in all transgenic lines (Table 3).

**Figure 7:**
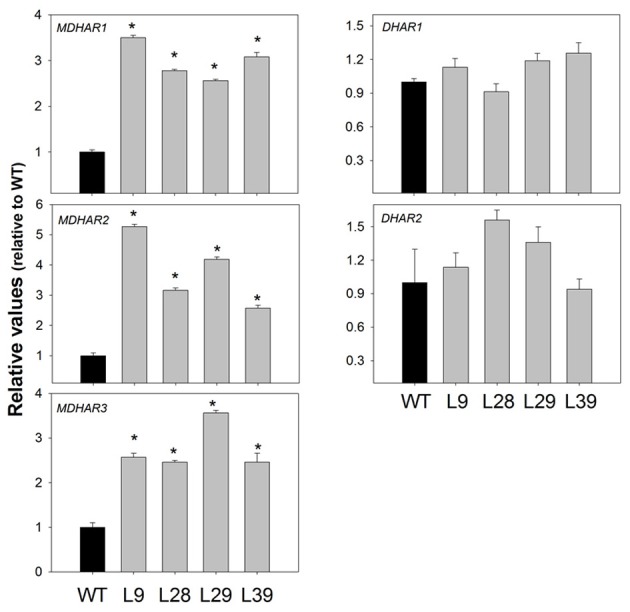
**Expression of *MDHAR*, and *DHAR* genes in red B33-PPi fruits**. The abundance of MDHAR1 (acc. number Solyc09g009390 ), MDHAR2 (acc. number Solyc02g086710), MDHAR3 (acc. number Solyc08g081530), DHAR1 (acc. number Solyc05g054760), and DHAR2 (acc. number Solyc11g011250). mRNAs were measured by quantitative RT-PCR, respectively. An asterisk indicates the values that were determined by the *t*-test to be significantly different (*P* < 0.05) from wild type.

Additionally, we also determined the gene expression of three monodehydroascorbate reductase (*MDHAR*) and two dehydroascorbate reductase genes (*DHAR*) found in tomato. Both are involved in ascorbate recycling pathway. Interestingly, all transformats displayed a significant increase in *MDHAR1, MDHAR2*, and *MDHAR3* transcript abundances (Figure 7). This result was in agreement with an increase in the MDHAR activity (Table 3). In contrast, we did not observed changes in the expression of the *DHAR1* and *DHAR2* genes (Figure 7).

## Discussion

Until now, the breeding of tomato has been dominated by a focus on traits that benefit the grower, such as yield, plant and fruit size, and storage characteristics (Schuch, 1994; Giovannoni, 2006; Cong et al., 2008). As a result, there has been a loss of consumer quality traits such as flavor and nutritional value, and this has focused recent interest on the molecular genetics of such traits (Giovannoni, 2001; Causse et al., 2002, 2004; Fraser et al., 2009; Mounet et al., 2009; Enfissi et al., 2010; Centeno et al., 2011; Morgan et al., 2013). The accumulation of a range of soluble metabolites is important for both flavor and nutrition. In this paper, we characterized the consequences of over-expressing an *E. coli* pyrophosphatase gene under fruit-specific promoter. This manipulation had a broad impact on fruit development and ripening, emphasizing both the important role of pyrophosphatase in ascorbate and starch biosynthesis.

### Effect of increasing pyrophosphatase activity on starch and sugars metabolism

Detailed analysis of sugars level revealed that starch content decreased while the major sugars, Suc and Glc increased in red ripe B33-PPi fruit. These data support the contention that active starch accumulation is an important contributory factor in determining the soluble solids content of mature fruit (Schaffer and Petreikov, 1997; Baxter et al., 2005). Here, we demonstrated that alterations in PPi metabolism have a strong effect on sugars metabolism and, hence, influence agronomic yield. Intriguingly, the data presented here are analogous to those previously described for transgenic potato plants in which higher PPi levels increased starch accumulation and decrease the level of Suc (Fernie et al., 2001a; Geigenberger et al., 2001), and decreased levels have been associated with lower starch biosynthetic rates (Geigenberger et al., 2001).

Different studies concerning starch metabolism in potato and tomato have suggested that AGPase activity plays an important role in its regulation (Geigenberger et al., 1999; Sweetlove et al., 1999). Regulation of the AGPase reaction has been very well characterized for several years. This enzyme is sensitive to allosteric regulation, being inhibited by inorganic phosphate and activated by 3PGA (3-phosphoglycerate) (Sowokinos, 1981; Sowokinos and Preiss, 1982). Additionally, it has been demonstrated to be transcriptionally regulated by sugars, nitrate, phosphate and trehalose-6-phosphate (Muller-Rober et al., 1990; Nielsen et al., 1998; Kolbe et al., 2005; Michalska et al., 2009). Moreover, it has been described that AGPase is also redox regulated (Tiessen et al., 2002; Centeno et al., 2011; Osorio et al., 2013) with malic acid potentially being a key component in this process at least in photosynthetically active tissues (Szecowka et al., 2012).

In this study, a strong correlation was found between starch concentration and AGPase activity in red ripe B33-PPi stage. Additionally, we observed that redox-state of AGPase was not altered in these transgenic fruit. This observation described here have lead us to propose that the activity of this enzyme was modified due to either a change in the rate of sugar influx into the tomato fruit and/or in the lower PPi levels found in these transgenic fruits.

### Increased activity of pyrophosphatase correlates with increased ascorbate content in tomato fruit

There is a large potential for improving ascorbate content in food products by means of both genetic engineering and breeding. The exploitation of the large natural variation in ascorbate content in many fruit crops gives the opportunity of improving their nutritional value by classical breeding. The generation of linkage maps and the conduction of quantitative trait loci (QTL) analysis allow the identification of genomic regions associated with ascorbate content (Davey et al., 2006; Stevens et al., 2007; Zorrilla-Fontanesi et al., 2011). Such QTL analyses therefore increase our knowledge of the molecular mechanism by which ascorbate is regulated in plants.

The strategy to improve the amount of ascorbate by genetic engineering has been based on the up-regulation of genes encoding for enzymes of the biosynthetic or recovery pathways. In general, plants transformed with genes from different pathways have shown variable increases in ascorbate content in different plant tissues (Agius et al., 2003; Chen et al., 2003; Tokunaga et al., 2005; Eltayeb et al., 2007; Badejo et al., 2008, 2009; Bulley et al., 2009, 2012; Hemavathi et al., 2009; Qin et al., 2011; Zhang et al., 2011; Cronje et al., 2012). Mean increases in ascorbate content were usually two- to three-fold i.e., similar to those reported here. Other approaches have used genes that do not encode enzymes of the ascorbate biosynthetic pathway in plants. Thus, the ectopic expression of a rat *L-gulonolactone oxidase*, a gene involved in the synthesis of ascorbate in animals, produced an increase of about 7-fold in lettuce (Jain and Nessler, 2000). Similar levels were observed in potato plants ectopically expressing a bacterial pyrophosphorylase or a yeast invertase (Farre et al., 2008).

In this study, a strong correlation was displayed between the cellular PPi and ascorbate levels (up to 2.5-fold increase in red ripe transgenic fruits) and it was demonstrated that this was mechanistically linked to pyrophosphatase activity as previously was observed in potato tuber overexpressing a bacteria pyrophosphatase with a plastid targeting sequence (Farre et al., 2006). Additionally, an increase in some intermediates of ascorbate biosynthesis such as dehydroascorbate, galacturonate, and *myo*-inositol were also observed.

Formation of GDP-D-mannose is the initial step in the D-Man/L-Gal pathway of ascorbate biosynthesis, which is synthetized from D-mannose-1 phosphate via GDP-mannose pyrophosphatase (Conklin et al., 1999; Keller et al., 1999) (Figure A1). This reaction in the direction of ascorbate biosynthesis produces PPi as by-product. It is thus conceivable that the removal of PPi is favorable for ascorbate synthesis. Furthermore, the observed higher expression of *GGP1, GGP2* genes, a key point for the control of ascorbate pathway (Dowdle et al., 2007; Laing et al., 2007; Bulley et al., 2012), and *GalDH*, the last gene in the AsA biosynthesis pathway, corroborates that D-Man/L-Gal pathway is activated in the B33-PPi fruits in comparison to WT fruits.

We also evaluated if other alternative pathways of AsA biosynthesis were altered in these fruits. When looked at the D-galacturonic acid pathway, an increase in the level of its precursor, galacturonic acid, was observed. However, the enzyme activities that catalyze the two last steps in this pathway, GalUR and aldonolactonase, were not altered. Increasing *myo*-inositol production has also shown varied results, with both increased (Lorence et al., 2004) and unaffected (Endres and Tenhaken, 2009) leaf AsA being reported. Together with an increase in *myo*-inositol levels, we observed a decrease in the *myo*-inositol oxygenase activity in the B33-PPi red fruits, suggesting that *myo*-inositol can act as a precursor for AsA biosynthesis as suggested by Lorence et al. (2004). Although, a previous study suggests that the L-galactose-1-phosphate phosphatase enzyme from the D-Man/L-Gal pathway, has a dual function that impacts both *myo*-inositol and AsA biosynthesis pathways (Torabinejad et al., 2009), further investigation are required to understand the impact of *myo*-inositol on AsA biosynthesis.

Also, our results revealed that ascorbate recycling pathway was altered in the B33-PPi red fruits since we found higher dehydroascorbic acid content and expression of the three tomato *MDHAR* genes.

### Increased activity of pyrophosphatase also affects other metabolic changes

When other areas of metabolism are considered some interesting observations are apparent. Interestingly, the total level of organic acids and amino acids were largely invariant in the B33-PPi lines in comparison with WT. The exception to this was that we observed an accumulation in two amino acids, namely Ala and Asp. The accumulation of Asp can be explained because it acts as precursor in the synthesis of Asn via asparagine synthase. This reaction produces PPi as by-product that can be removed via pyrophosphatase. Therefore, we expect a shift in the reaction equilibrium to favor the Asn synthesis direction. Although significant differences were not found in the levels of Asn, this may be due to a co-ordinate up regulation of its metabolism. The reason for the increase in alanine is less clear but may merely reflect the additional availability of glucose for glycolytic reactions.

### Morphological effects on B33-PPi fruits

In addition to the increase in soluble solids content, the fruit of the transgenic lines were compromised in size. This observation was also largely to be expected, since several direct genetic studies (Zrenner et al., 1996; Sonnewald et al., 1997; Sturm and Tang, 1999) have implicated Suc mobilization as a key determinant of sink strength in a broad range of species. As much as 10% (w/v) Suc has been reported in the phloem of plants (Hayashi and Chino, 1990), and the presence of AsA in the phloem sap was confirmed by radiolabeling (Franceschi and Tarlyn, 2002). It was also reported that the presence of sugar within the plant acts as potent signal that promotes AsA biosynthetic gene expression (Nishikawa et al., 2005). Within tomato fruit itself, a positive correlation between Suc feeding and the expression level in some genes of the D-Man/L-Gal pathway was described (Badejo et al., 2012), supporting the view that transportation of sugars from source tissues affect the AsA content in sink tissues through the up-regulation of AsA biosynthesis pathway genes. Despite the wide changes in morphological parameters, metabolic changes in the transgenic fruit were, by and large, confined to sugar and AsA metabolisms. We believed that fruit growth is largely dependent on the relationship between import of photoassimilate and AsA intake and/or biosynthesis.

In summary, the results presented in this study provide direct evidence that the reduction in PPi content had strong effects on metabolism of sugar and ascorbate contents. Detailed analysis of starch metabolism revealed that this phenomenon was due to alteration in AGPase activity, caused by either a change in the rate of sugar influx into the tomato fruit and/or in the lower PPi levels found in these transgenic fruits. During ripening, the lack of accumulation of transitory starch was reflected by a decrease of soluble sugars. Moreover, we demonstrated that alterations in the level of PPi resulted in dramatic effect on ascorbate metabolism. These lines displaying low PPi content showed and increased flux to, and accumulation of, ascorbate. This occurred in spite of increases the ascorbate level via D-mannose-1P and via GDP-mannose pyrophosphatase. Further investigation is required to define this control, especially in fruit such as tomato, where it may contribute to taste (sugars and organic acids) and nutritional value (ascorbate) of the fruit which are important in determining fruit quality.

## Materials and methods

### Plant material

The gene encoding a pyrophosphatase from *E. coli* (Sonnewald, 1992) was introduced in the sense orientation into the vector pBinAR between the patatin B33 promotor (Rocha-Sosa et al., 1989) and the octopine synthase polyadenylation signal. This construct was introduced into tomato (*Solanum lycopesicum*, L.) cv Moneymaker plants by an Agrobacterium-mediated transformation protocol, and plants were selected and maintained as described in the literature (Tauberger et al., 2000). An initial screening was carried out on the basis of pyrophosphatase activity. This screen allowed the identification of four lines, which were taken to the next generation.

### Metabolite determinations

#### PPi and Pi determination

PPi was extracted from tomato fruit by TCA/ether method (Jelitto et al., 1992). PPi was determined using the colorimetric PiPer Pyrophosphate assay kit (Invitrogen) according to the manufacturer's specifications. All glassware was pretreated overnight with 0.1 M HCl to remove residual phosphate. PPi levels were determined by a sample blank with or without sPPase, and total Pi was calculated by comparison at 595 nm with a linear Pi standard curve.

Pi was determined in the TCA extracts with a colorimetric assay as described by Taussky and Shorr (1953).

#### Primary metabolic profiling

Metabolite extraction derivatization, standard addition, and sample injection for GC-MS were performed according (Osorio et al., 2012). Both chromatograms and mass spectra were evaluated using TAGFINDER (Luedemann et al., 2008).

#### Ascorbic acid determination

Ascorbic acid extraction and analysis were performed as described (Lima-Silva et al., 2012). Ascorbic acid content was determined by comparison with a linear ascorbic acid standard curve.

#### Starch determination

The level of starch in the tissues were determined exactly as described previously (Fernie et al., 2001b).

### Enzyme activities

#### Alkaline pyrophosphatase activity

The protein extraction and enzyme activity were analyzed as described Farre et al. (2001).

#### AGPase

AGPase activity was measured in the pyrophosphorolysis direction with a spectrophotometric assay, as described Tiessen et al. (2002, 2003). Frozen tissues were homogenized in liquid N_2_ and approx. 100 mg was extracted rapidly (1 min) with 1 ml of extraction buffer (50 mM Hepes-KOH, pH 7.8, and 5 mM MgCl_2_) at 4°C. After centrifugation (30 s at 13,000 g at 4°C), 10 μl of the supernatant was used for the AGPase assay. The reaction was performed in a total volume of 200 μl containing 50 mM Hepes-KOH, pH 7.8, 5 mM MgCl_2_, 10 μM Glc-1,6-bisP, 0.6 mM NADP^+^, 2.5 mM Na-PPi, 1 unit/ml phosphoglucomutase, 2.5 units/ml Glc-6-P dehydrogenase, and a range of concentrations of ADP-Glc (0.4−1 mM) in the absence of Pi, with or without DTT (10 mM) for activation assay. Reactions were followed on line at 340 nm and were linear up to 30 min. The activation state of AGPase is defined as the ratio of Vsel (−DTT) to Vred (+DTT).

#### Myo-inositol oxygenase

Two-hundred mg of tissue was incubated for 30 min at 30°C in a buffer containing 100 mM KPO_4_ (pH 7.2), 2 mM L-cysteine, 1 mM ammonium ferrous sulfate hexahydrate, and 60 mM *myo*-inositol. The reaction was stopped by boiling for 10 min and denatured protein removed by centrifugation (20,000 g, 15 min). Glucuronic acid was determined at 540 nm before and after samples developed a pink color with addition of a 3-hydroxybiphenylphenol color reagent (van den Hoogen et al., 1998).

#### D-galacturonate reductase

One gram of samples were homogenized in liquid nitrogen and extracted with 50 mM sodium phosphate buffer, pH 7.2, containing 2 mM EDTA, 2 mM dithiothreitol, 20% glycerol and PVPP. GalUR activity was measured by the decrease in absorbance at 340 nm at 25°C after the addition of 100 μ l of crude enzyme extract to the assay mixture (1 ml) consisted of 50 mM phosphate buffer (pH 7.2), 2 mM EDTA, 0.1 mM NADPH, 30 mM D-galacturonic acid and 2 mM dithiothreitol. The GalUR activity in the crude enzyme extract was recorded as nmol of NADPH oxidized min-1 mg-1 protein (Agius et al., 2003).

#### Aldonolactonase

The activity was measured by the change in absorbance of *p-nitrophenol* through acidification at 405 nm essentially as described Ishikawa and Shigeoka (2008).

#### L-galactono-1,4-lactone dehydrogenase

Samples were prepared as described by Mieda et al. (2004) and assayed at 340 nm by measuring the reduction of NAD^+^ in a reaction mixture containing 0.5 mM NAD^+^, 1 mM L-Gal, and the enzyme extract. L-Galactono-1,4-lactone dehydrogenase activity was assayed by the reduction of cytochrome *c* resulting in an increase in absorbance at 550 nm in a reaction mixture containing 50 mM TRIS-HCl, pH 8.5, 1 mM sodium azide, 42 mM L-Gal, 0.1% Triton X-100, 1.05 mg^−1^ ml cytochrome *c*, and the extract in a final volume of 1 ml as described by Yabuta et al. (2000).

#### Monodehydroascorbate reductase

The activity was measured according to the method of Hossain and Asada (1984).

### Measurement of fruit brix

Ripe fruit tissue was homogenized with a razor blade, and the soluble solids (Brix) content of the resulting juice measured on a portable refractometer (Digitales Refrktometer DR6000; Krüss Optronic GmbH, Hamburg, Germany).

### Analysis of gene expression by QRT-PCR

Total RNA was extracted according to Bugos et al. (1995) with minor modifications. Integrity of the extracted RNA was checked by electrophoresis under denaturing conditions after treating the RNA with RNase-free DNaseI (Roche). First-strand cDNA synthesis of 1 mg of RNA in a final volume of 20 mL was performed with Moloney murine leukemia virus reverse transcriptase, Point Mutant RNase H Minus (Promega), according to the supplier's protocol using oligo(dT) T19 primer.

Expression of the monodehydroascorbate reductase (*MDHAR*), dehydroascorbate reductase (*DHAR*), L-galactono-1,4-lactone dehydrogenase (*GaLDH*), and L-galactose dehydrogenase (*GDH*) genes was analyzed by real-time qRT-PCR using the fluorescent intercalating dye SYBR Green in an iCycler detection system (Bio-Rad; http://www.bio-rad.com/). Relative quantification of the target expression level was performed using the comparative Ct method. The following primers were used: for analysis of *MDHAR1* transcript levels (GenBank accession no. Solyc09g009390), forward, 5′-TCTACGGTGATAATGTGGGTGA-3′, reverse, 5′-ATTGCCTTGTTCTCTTCAGGTG-3′; for *MDHAR2* (GenBank accession no. Solyc02g086710), forward, 5′-TTGAGTGATAAACCAGAGCCATC-3′, reverse, 5′-TTCTACGCCTCCTACCATACCA-3′; for *MDHAR3* (GenBank accession no. Solyc08g081530), forward, 5′-ATTTCAAGGGTTTCGGTTCCT-3′, reverse, 5′-CATTTCCTCCTCCAACTACCAC-3′; for *DHAR1* (GenBank accession no. Solyc05g054760), forward, 5′-TTTCCTACCTTCGTCTCATTTCTG-3′, reverse, 5′- GAACAAACATTCTGCCCATTGA -3′; for *DHAR2* (GenBank accession no. Solyc11g011250), forward, 5′-GCTTCATTTGCGACTTCTATCAA-3′, reverse, 5′-AAAACCTCTTCTGGGTGCTCTG-3′; for *GaLDH* (GenBank accession no. Solyc10g079470), forward, 5′-GCTATTTCGGTATGCTCCGTTG-3′, reverse, 5′-CCTCACATTCGCTTCTTTCACT-3′; for *GDH* (GenBank accession no. Solyc01g106450), forward, 5′-TGTTTGTCAGTTCAACGAGGTC-3′, reverse, 5′-TTGTTTTAGATGTCCAAGTGCAA-3′ (Gilbert et al., 2009); for *GGP1* (GenBank accession no.Solyc06g073320) forward, 5′-AGGGTGCAACTGAGGCAAATGC-3′, reverse, 5′-ATGGGCTGTGGAGGTGTGACA-3′; for *GGP2* (GenBank accession no.Solyc02g091510) forward, 5′-GTCTTGGTTGGAGGTTGTAAT-3′, reverse, 5′-TGCACAAAAGTTGCTAGTCCT-3′. To normalize gene expression for differences in the efficiency of cDNA synthesis, transcript levels of the constitutively expressed elongation factor 1a of tomato (GenBank accession no. X14449) were measured using the following primers: forward, 5′-ACCACGAAGCTCTCCAGGAG-3′, reverse, 5′-CATTGAACCCAACATTGTCACC-3′ (Zanor et al., 2009).

### Conflict of interest statement

The authors declare that the research was conducted in the absence of any commercial or financial relationships that could be construed as a potential conflict of interest.
